# Effects of high-intensity circuit training, low-intensity circuit training and endurance training on blood pressure and lipoproteins in middle-aged overweight men

**DOI:** 10.1186/1476-511X-12-131

**Published:** 2013-09-03

**Authors:** Antonio Paoli, Quirico F Pacelli, Tatiana Moro, Giuseppe Marcolin, Marco Neri, Giuseppe Battaglia, Giuseppe Sergi, Francesco Bolzetta, Antonino Bianco

**Affiliations:** 1 Department of Biomedical Sciences (DSB), University of Padova, Via Marzolo 3, Padova 35131, Italy; 2 Italian Association of Medicine and Fitness (AIFeSM), Ravenna, Italy; 3 Dept. of Sports and Exercise Science (DISMOT), University of Palermo, Palermo, Italy; 4 Department of Medicine (DIMED)- Geriatric Unit, University of Padova, Padova, Italy

**Keywords:** Circuit training, Exercise, Cardiovascular disease risk factors, Lipoproteins, Hdl, Ldl, Triglycerides, Blood pressure

## Abstract

**Background:**

The aim of this study was to determine the physiological effects of an high-intensity circuit training (HICT) on several cardiovascular disease risk factors in healthy, overweight middle-aged subjects, and to compare the effects of HICT to traditional endurance training (ET) and low-intensity circuit training (LICT).

**Methods:**

Fifty-eight participants (ages 61±3.3 yrs, BMI 29.8±0.9) were randomly assigned to one of the three exercise treatment groups: HICT, LICT and ET. The three groups exercised three times per week, 50 min per session for 12 weeks. Baseline and after intervention anthropometric characteristics: body weight (BW), fat mass (FM); blood pressure: diastolic (DBP) and systolic (SBP), blood parameters; CHOL-t (total cholesterol), LDL-C (low density lipoprotein-cholesterol), HDL-C (high density lipoprotein-cholesterol), TG (triglycerides), ApoB and ratio ApoB/ApoA1 were measured.

**Results:**

Compared to other groups, HICT showed significantly higher reductions in FM, DBP, CHOLt, LDL-C, TG, ApoB and significantly greater increases in high density HDL-C. LICT resulted in the greatest reduction in SBP. All groups showed a significant improvement of BW without any significant differences between groups.

**Conclusions:**

Our findings indicate that high-intensity circuit training is more effective in improving blood pressure, lipoproteins and triglycerides than endurance training alone or lower intensity circuit training.

## Background

Regular exercise is known to have a positive effect on many cardiovascular disease risk factors [1]. Whether its benefits lie in the fact that it helps to control weight, improve the body’s ability to use insulin, condition the heart muscle, increase levels of protective HDL cholesterol, moderate stress, lower blood pressure, or a combination of these effects, is not clear. Whatever the reason, it is generally accepted that regular exercise can lower cardiovascular risk and it should be encouraged for everyone within the limits of each individual [1]. Both aerobic endurance exercise and resistance training can promote substantial benefits in physical fitness and health-related factors in older individuals [2-4]. Endurance training seems to be more effective in decreasing fat mass, resting heart rate and blood pressure [1] whereas resistance training seems to be more effective for enhancing bone mineral density [5] muscle strength [6], basal metabolism [7,8] and muscle and connective tissue cross-sectional area [9]. Both training methods have been found to improve glucose metabolism and lipid profile even if with conflicting results [10-12]. Aerobic endurance training has a greater impact on maximum oxygen uptake (VO_2_max) and associated cardiopulmonary variables, and it more effectively modifies cardiovascular disease risk factors associated with the development of coronary artery disease [13]. On the other hand regular resistance training offers the greatest potential for developing muscular strength, endurance, and mass. It also assists in the maintenance of basal metabolic rate (to complement aerobic training for weight control) [8]. Although the mechanisms for improvement may be different, both endurance and resistance training appear to have similar effects on bone mineral density, glucose tolerance, and insulin sensitivity [14-17].

Considering the above factors, some researchers suggested that a circuit-based training consisting of endurance and resistance exercises might be preferred, rather than one focused only on a single mode of exercise [18,19] even if not all researchers agreed [20,21]. Regarding the better intensity there is no accordance between researchers: even if public health recommendations have emphasized the value of moderate intensity aerobic exercise for improving cardiovascular health and reducing the risk of coronary heart disease (CHD) [15] recent studies suggest that the higher the exercise intensity, the greater the increase in aerobic fitness [22,23].

To our knowledge, no data are available on the effects of circuit training performed with different intensity both during aerobic and strength exercises on blood pressure and blood lipids in middle-aged men. In our previous preliminary study we have demonstrated that a high intensity circuit training is more effective in improve strength and body composition [19]. The purpose of the present investigation, was to compare three different exercise protocols on cardiovascular disease risk factors in healthy overweight adult men. In particular, we aimed to examine the possible beneficial effects of high-intensity circuit training composed of resistance and aerobic exercise on blood lipids and blood pressure in sedentary middle-aged subjects (60–65 years).

## Methods

### Subjects

Healthy, untrained 61±3.3 year-old men were recruited for the investigation by poster and mail advertising. Subjects with no contraindications to experimental procedure were invited for a clinical examination. Respondents provided informed consent to participate in the study and were screened for the presence of diseases or conditions that would place them at high risk for adverse responses to exercise. Inclusion criteria are BMI up to 25 kg/m^2^, age 55> yrs and <70. Exclusion criteria for the study included history of recent myocardial infarction, severe cardiac arrhythmia, unstable angina, poorly controlled hypertension, poorly controlled diabetes mellitus, frequent or complex ventricular ectopy, or significant cognitive dysfunction that might interfere with one’s ability to adhere to exercise protocols [24]. Moreover, subjects in the inflammatory stage of arthritis or those receiving medical treatment for osteoporosis were also excluded from the study. Subjects’ basal characteristics were descripted in Table 1.

**Table 1:** Representative cardio-vascular risk factors of subjects at baseline time point

		**HICT**	**LICT**	**ET**	***p value***
		**Pre-test**	**Pre-test**	**Pre-test**	*n.s*
BW (kg)		89.4±2.7	86.8±4.4	87.6±4.6	*n.s*
FM (Kg)		30.9±8.2	30±9.0	29.7±7-1	*n.s*
LBM (Kg)		58.5±8.2	56±7.7	57.9±5.9	*n.s*
DBP (mm/Hg)		85±6	82±5	83±7	*n.s*
SBP(mm/Hg)		138±12	136±10	140±8	*n.s*
TG	(mmol/L)	2.66±0.05	2.66±0.04	2.61±0.03	*n.s*
	*(mg/dL)*	*(235*±4)	*(235±3.2)*	*(231.7±2.9)*	
CHOLt	(mmol/L)	5.52±0.1	5.88±0.09	5.6±0.1	*n.s*
	*(mg/dL)*	*(213*±3.7)	*(227±3.6)*	*(216.3±4)*	
HDL-C	(mmol/L)	1.32±0.02	1.3±0.03	1.28±0.04	*n.s*
	*(mg/dL)*	*(51±0.6)*	*(50.2±1.2)*	*(49.3±1.6)*	
LDL-C	(mmol/L)	2.98±0.1	3.05±0.12	3.12±0.11	*n.s*
	*(mg/dL)*	*(115*±3.8*)*	*(117.6±4.5)*	*(120.6±4.2)*	
ApoB/A1		0.73±0.12	0.75±0.14	0.77±0.18	*n.s*

*Legend: BW* body weight, *FM* fat mass, *LBM* lean body mass, *DBP* diastolic blood pressure, *SBP* systolic blood pressure, *TG* triglycerides, *CHOLt* total cholesterol, *HDL-C* high density lipoprotein-cholesterol, *LDL-C* low density lipoprotein-cholesterol, *ApoB* apolipoprotein B*. p value* set at .05. Triglycerides and blood lipids values are also showed (in Italic) also as mg/dL.

### Study design

In an initial interview each respondent was provided all details about the treatment protocol. All subjects gave their informed consent and the study was approved by the Ethical Commission of the Department of Anatomy and Physiology of the University of Padova.

After providing informed consent, the participant was interviewed regarding his or her health status. A modified version of the Health Status Questionnaire [25] was used as a screening tool. A 1-yr physical activity recall questionnaire for older adults [26] was also completed during this initial visit. This activity inventory inquires as to the number of hours per week spent performing various activities from household duties to more vigorous activities. After completion of the above, each respondent was further evaluated by physical examination and maximal graded exercise test with electrocardiography during a cardiological evaluation. This was the maximal test’s protocol: following 3 min of baseline cycling at 20 W, work-rate was increased by 1 W every 3 s (i.e. 20 W/min) until the participant was unable to continue despite encouragement. The participants cycled at a self-selected pedal rate which remained constant throughout the test (70–90 rev/min). Heart rates were continuously monitored with electrocardiography. Participants were randomly assigned to one of three treatment groups (described in detail below): an Endurance Group (ET n=20), a Circuit Low intensity Group (LICT n=19), or a Circuit High intensity Group (HICT n=19). Subsequently, the participants trained three times per week, 50 min pre session for a period of 12 weeks, after which the submaximal tests were repeated.

### Experimental procedures

Weight was measured to the nearest 0.01 kg using an electronic scale, and height to the nearest 0.01 m using a Harpenden portable stadiometer. Functional assessment was conducted using a 100 Watt constant test for 15’ (70 rpm) cycle ergometer assessing FC at the 15th minute using a Polar heart rate monitor (Polar S810; Polar, Kemple, Finland) and blood lactate using SensLab Lactate Scout – Test strips (Bautzner Staβe 67; Leipzig, Germany) based on the blood lactate system oxidised with redox reaction via electrode mediation. Peak blood lactate concentration 3 min after cessation of exercise was measured to verify metabolic commitment. During the maximal incremental test descripted above the maximal VO_2_ was also measured. During the exercise test the subjects breathed trough a face mask for a breath-by-breath analysis of oxygen uptake (V’O_2_), carbon dioxide production (V’CO_2_) using an Ergocard® ergospirometer (Pacific Medical Systems, Hong Kong, S.A.R) and a ‘pitot tube’ pneumotacograph equipped with standard gas analysers. The system was calibrated before each measure using calibration syringes and precision oxygen and carbon dioxide gas mixtures. Subjects were requested to abstain from caffeine or alcohol consumption for 24 h prior to the measurement.

A familiarization session was performed before the tests. Tests were performed in the morning after a time of two hours from the same light breakfast; the subjects did not have any intense physical activity the day before measurement.

Blood analyses were done for total cholesterol (CHOLt), triacyglicerol (TG) high-density lipoprotein cholesterol (HDL-C), low-density lipoprotein cholesterol (LDL-C), apolipoprotein B (ApoB), apolipoprotein A1 (ApoA1), both at the start of the experiment and after the end of the 12^th^ week of training. None of the subjects had exercised the day before blood samples were obtained. Before and after the training period, blood samples were taken at 8 A.M. at rest and after an overnight fast. To avoid interassay variation all blood samples were stored at −80° and analysed together at the end of the study in a certified laboratory. Fasting total cholesterol, HDL-C, LDL-C, and TG were measured by an enzymatic colorimetric method using a Modular D2400 (Roche Diagnostics, Basel, Switzerland). Serum apoB and apoA1 concentrations were determined by an immunoturbidimetric method using a Roche/Hitachi Modular P analyzer (Roche Diagnostics, Basel, Switzerland). LDLc fraction was calculated from Friedewald’s formula: LDLc = TC – HDLc – (TG/5) [27]. Fat-free mass (FFM) and fat mass (FM) were established by Dual Energy X-ray Absorptiometry (DEXA) with fan-beam technology (Hologic QDR 4500W, Inc.). The DEXA is the most reliable method for assessing regional body composition, because it is very straight-forward, safe and suitable for people who are elderly or ill, the radiation dose required is minimal and the measurements obtained are generally more precise than those obtainable using other techniques [28]; the reproducibility of the total body composition measurements is accurate in consecutive tests [28,29].

### Diet

No changes were made in the diets of the three groups in order to avoid entering another variable into the training protocol. Checks were made to make sure that none of the men had experienced a loss of weight of more than ± 0.3 kg in the past few months. The diet of the three groups analysed using DietNext® (GSA Tea, Caldogno, VI, Italy) software demonstrated a substantial similarity: 63% carbohydrates, 17% proteins, 20% fats (equally divided in monounsaturated, polyunsaturated and saturated fat). Energy intake was 10,850.5±653,2; 10,635±710.3; 10,927±786.4 kJ/day for HICT, LICT and ET respectively. The mean calories are divided during the day as following: breakfast (21% of daily calories), lunch (35% of daily calories), dinner (37% of daily calories), the remaining calories were assumed during morning or afternoon snack. In addition, to verify that none of the subjects modifies the nutritional behaviour during the intervention protocol an assessment of current dietary intake was performed using the Italian version of the food frequency questionnaire developed and validated in the context of the European Prospective Investigation into Cancer and Nutrition (EPIC) [30]. The questionnaire confirmed that subjects maintained the same diet regards percentage of nutrients and Kj/day.

### Training protocols

The participants trained 3 times a week (with at least 1 day of rest between session) and 50 min per session for 12 weeks under the supervision of qualified trainers with degrees in Human Movement Sciences and supervised by the researchers. The subjects to instructor ratio was less than 6 to 1, so the instructors were able to monitor RPE, HR and technical execution during training sessions. Training sessions were conducted in a Fitness Gym.

To set up the intensity of aerobic training we have used the maximum HR (Heart Rate) obtained during the maximal graded test; during training, to asses exercise intensity, we used the Karvonen’s formula [31] to establish the HRR (Heart Rate Reserve).

Each session included a warm-up and cool-down period involving 5 min of low-intensity walking and light stretching activities.

Before began experimental diversified training protocol each subject underwent a 2 week familiarization training consisting in 30’ of endurance session on cycloergometer at 60% HRR and after 5 exercises for each muscle group of 2 sets of 15 reps.

Subjects performed a 6-RM test on the different exercises included in the training schedule as described elsewhere [8]. A 6 RM test is suitable to test maximal strength in subjects with little or no previous resistance training experience. Data obtained from initial test was used to determine an appropriate starting resistance training. This technique has been shown to have a high reproducibility (r=0.99).

The subjects were divided into the following 3 groups:

1. ET: these participants trained on cycloergometer. Intensity was maintained at 50% of HRR. The initial duration of the endurance work was 30 min per session, and the duration was increased by 3 min·wk ^-1^ until a maximum of 40 min was achieved and maintained throughout the remainder of the program. At the end of central part of training, subjects performed 4 sets of 20 reps of abdominal crunches to increase the attractiveness of this kind of training.

2. LICT: the participants trained by alternating 8’ of endurance on cycloergometer at 50% of HRR with training at resistance exercise stations (back: underhand cable pulldowns; chest: pectoral machine; shoulders: lateral shoulder raise; lower limbs: horizontal press, the exercises were performed to reach 15 reps maximum; abdomen: 1 set of 20 reps abdominal crunches. Subjects wait 60” between one exercise and the following. After concluded one complete round subjects repeat all another two times.

3. HICT: the participants trained by alternating 8’ of endurance on cycloergometer (performed for 3’ at 50% and 1’ at 75% of HRR), with training at resistance exercise stations. Back: underhand cable pulldowns; chest: pectoral machine; shoulders: lateral shoulder raise; lower limbs: horizontal press; abdomen: 1 set of 20 reps abdominal crunches at the end of each resistance exercise station performed with 3 sets of Rest Pause. Rest pause is a methodology of training that involved a very high intensity of training [8,19,32]. Every set performed with Rest Pause technique consists in: 6 RM, 20” recovery, two reps at exhaustion 20” recovery, one or two more reps until exhaustion has been reached. Subjects wait 60” between one exercise and the following. After concluded one complete round subjects repeat all another two times. We defined high exercise intensity 75% of HRR and low intensity 50% of HRR according to Balady et al. [33].

### Statistical analysis

Multivariate analysis of variance (MANOVA) on difference measures was used to compare the mean ages, heights, weights, physical activity scores across all groups. Whenever significant differences in values occurred, multiple comparisons test (useful for determining where significant differences occur between pairs of groups) was performed using a post-hoc Tukey-Kramer test, considered the most powerful method for all pairwise comparisons. Alpha significance level was set at 5% (and was adjusted for multiple comparisons). Statistica Software, ver. 8.0 (Tulsa, USA) was used for the analysis.

## Results

Of the 78 respondents, 60 passed screening and elected to continue with the study. Two subjects did not reach the 80% attendance (one in ET and one in HICT). Therefore, the data presented below are from 58 subjects. None of the drop out left the program as a result of injuries or adverse responses to the treatment. Finally HICT and LICT groups included 19 subjects, while ET consisted of 20 individuals. There were no significant differences at the baseline between the three groups with regards to all variables taken into account (Table 1). Moreover the groups did not differ in number of sessions attended.

Values of the parameters which were taken as representative cardio-vascular risk factors measured before and after the 12 weeks of training are reported in Table 2. As showed in Table 2, body-weight was significantly reduced in HICT and LICT compared to ET whereas HICT showed greater and significant reduction in %FM than LICT and ET. We found a greater reduction of DBP in HICT compared to LICT and ET. On the contrary LICT showed a great decrease in SBP compared to HICT and ET. HICT group showed a significant decrease in CHOLt and LDL-C compared to LICT and ET, but both LICT and ET showed a significant reduction. HICT showed a significant increase of HDL-C compared to other groups whilst there are no significant changes of this parameter in LICT and ET. Moreover, there was a greater significant decrease of TGs in HICT group compared to other two groups. ApoB/ApoA1 ratio decrease significantly in all groups without any significant differences between samples.

**Table 2:** Representative cardio-vascular risk factors measured before and after the 12 weeks of training

		**HICT**			**LICT**			**ET**		
		**pre**	**post**	***p***	**pre**	**post**	***p***	**pre**	**post**	***p***
BW (kg)		89.4±2.7	86.3±2.3	p<0.001 * °	86.8±4.4	84.2±3.7	p<0.05	87.6±4.6	84.4±4.1	p<0.05
FM (Kg)		30.9±8.2	25.5±6.3	p<0.001 *°	30±9.0	27.1±6.5	p<0.05	29.7±7-1	28.2±6.5	p<0.05
LBM (Kg)		58.5±8.2	60.8±7.3	p<0.005 *°°	56±7.7	57.1±6.9	p<0.05 #	57.9±5.9	56.2±6.4	n.s.
DBP (mm/Hg)		85±6	79±7	p<0.001 *°	82±5	80±6	p<0.05	83±7	80±5	p<0.05
SBP(mm/Hg)		138±12	131±9	p<0.05	136±10	125±9	p<0.001 * #	140±8	135±11	p<0.005
TG	(mmol/L)	2.66±0.05	2.26±0.02	p<0.001*°	2.66±0.04	2.46±0.04	p<0.005	2.61±0.03	2.53±0.05	p<0.005
	*(mg/dL)*	*(235*±4)	*(200*±1.7)		*(235±3.2)*	*(218.8±3.4)*		*(231.7±2.9)*	*(224.2±4.3)*	
CHOLt	(mmol/L)	5.52±0.1	5.0±0.06	p<0.005 *°	5.88±0.09	5.73±0.09	p<0.05	5.6±0.1	5.44±0.11	p<0.05
	*(mg/dL)*	*(213*±3.7)	*(193*±2.4)		*(227±3.6)*	*(221±3.6)*		*(216.3±4)*	*(210.9±4.3)*	
HDL-C	(mmol/L)	1.32±0.02	1.45±0.03	p<0.001 *°	1.3±0.03	1.33±0.04	n.s	1.28±0.04	1.27±0.05	n.s.
	*(mg/dL)*	*(51±0.6)*	*(56±1.2)*		*(50.2±1.2)*	*(51.2±1.4)*		*(49.3±1.6)*	*(49.1±1.8)*	
LDL-C	(mmol/L)	2.98±0.1	2.51±0.08	p<0.05 *°	3.05±0.12	2.95±0.12 #	p<0.05	3.12±0.11	3.03±0.12	p<0.05
	*(mg/dL)*	*(115*±3.8*)*	*(*97±3.2)		*(117.6±4.5)*	*(114.4±4.8)*		*(120.6±4.2)*	*(116.9±4.7)*	
ApoB/A1		0.73±0.12	0.65±0.1	p<0.05	0.75±0.14	0.69±0.12	p<0.05	0.77±0.18	0.70±0.14	p<0.05

*Legend: BW* body weight, *FM* fat mass, *LBM* lean body mass, *DBP* diastolic blood pressure, *SBP* systolic blood pressure, *TG* triglycerides, *CHOLt* total cholesterol, *HDL-C* high density lipoprotein-cholesterol, *LDL-C* low density lipoprotein-cholesterol), *ApoB* apolipoprotein B. * =p<0.05 HICT vs LICT, ° = p<0.05 HICT vs ET; °°=p<0.001 LICT vs ET; #=p<0.05 LICT vs ET. Triglycerides and blood lipids values are also showed (in Italic) also as mg/dL.

## Discussion

The main finding of this study was the greater effect on blood lipids improvement of a high intensity circuit compared to a lighter circuit or to an endurance training. Moreover different kinds of circuit showed different effects on blood pressure. A low intensity circuit training more improved SBP compared to high intensity training and endurance training, while the effect of exercise on DBP was greater in response to high intensity circuit training. Although resistance training has long been accepted as a means for developing and maintaining muscular strength, endurance, power, and muscle mass (hypertrophy) [34,35], its beneficial relationship to health factors and chronic disease has only recently been recognized in the scientific literature [36,37]. Prior to 1990, resistance training was not a part of the recommended guidelines for exercise training and rehabilitation for either the American Heart Association or the American College of Sports Medicine (ACSM). In 1990, the ACSM recognized resistance training as a significant component of a comprehensive fitness program for healthy adults of all ages [38], position subsequently confirmed few years after [39].

Regarding the role of the exercise’s intensity on health parameters despite the increasing interest on high intensity training there is still no accordance between researchers. Clinical trials generally reported greater improvements after vigorous (e.g. ≥ 60% aerobic power i.e. 60% HR res or 75% HR max) compared with moderate intensity (e.g. between 50 and 60% aerobic power) exercise for diastolic blood pressure, glucose control and aerobic capacity, but reported no intensity effect on improvements in systolic blood pressure, lipid profile, or body fat loss [23].

Starting from this last parameter, in the present study, we observed greater weight and fat loss in HICT group. To explain this result, we can argue that such kind of training could affect positively EPOC. As a matter of fact intensity and duration of exercise appears to have a key role to determine the increase in EPOC after a high intense training and multiple mechanisms potentially underlie this effects [8]. As we have previously shown, they include, at least, an increase in resting energy expenditure (REE) and a reduction in respiratory ratio (RR) [8] even if other different mechanisms are supposed to be involved. The suggested other pathways include protein re-synthesis due to post-exercise muscle damage (that, has been calculated, contribute 20% increase in resting metabolism) [40,41], glycogen metabolism [42], AMPK/ACC (AMP kinases/ Acetyl CoA Carboxylase) pathway [43], ANP (atrial natriuretic peptide) production [44,45] and cytokines effects [46]. All the above mentioned actors should be related to the intensity of exercise [47]. The effects of different kind of exercise deserve some comments. Some research suggested that RT seems to be more effective on obesity and systolic blood pressure [17] but the precise amount and intensity of this multifaceted methodology of exercise are still not well known. The reason could be due to different modalities of resistance training execution. RT include a lot of variables, such as speed of movement both in eccentric and concentric phase, number of set and repetitions, recovery, different kind of exercise, etc., that make difficult to study this kind of training and could explain the contradictory results and guidelines indications [34,35].

Recent results confirm that both dynamic RT and static RT have a beneficial effect both in subjects with optimal pressure and/or prehypertension. Indeed, a dynamic resistance exercise training significantly decreases BP, increases peak VO_2_, and decrease body fat and plasma triglycerides [48] Regarding potential risks, a recent paper by Moro et al. [49] showed that an high intensity resistance training has the same effect on arterial blood pressure than the commonly proposed protocols to cardiovascular patients and suggested by ACSM’s guidelines. As far as concerns lipids profile, in this work we observed that HICT group showed a significant decrease in CHOLt, TG and LDL-C and an increase in HDL-C compared to LICT and ET. It has been specifically demonstrated that cholesterol and triglyceride levels can be improved through regular resistance training in middle-aged subjects [50,51] and training is associated with a decrease in total and abdominal fat as well as an increase in HDL-cholesterol in the elderly [52]. As suggested for fat loss exercise intensity might be a key factor in blood lipids’ exercise response. There are indeed some evidences that total energy expenditure could be related to changes in lipids and lipoproteins in a dose–response manner; in other words changes in blood lipid and lipoprotein concentration seem to depend on total amount of calories expended [53]. Our results are in agreement with those of Dalleck and colleagues [54], confirming the hypothesis [55,56] of a positive effect of RT on blood lipids variables even if are in contrast with Wooten’s findings who found instead no changes in HDL-c after 12 weeks of resistance training [57]. A recent systematic evidence suggests that RT could positively affects LDL- c, while combined exercise (as our circuit training) show improvements of both HDL and LDL cholesterol and high-intensity endurance training improves HDL-c. [58] but with conflicting results that could be attributed to the above mentioned variables of RT. During exercise, an increase in lipoprotein lipase activity, responsible for triglycerides breakdown, has been proposed as a molecular cause for HDL-C increase [59] PPAR-ϒ and PGC1-α could be also implied in the possible mechanisms that underlies the improvement in lipid profile [60] but the precise mechanism involved has been not, at the moment, clarified.

There are several strength and, obviously, limitations that should be considered with respect to our study’s outcomes. To our knowledge, this is the first study aimed to investigate the effects of a high-intensity interval circuit training on cardiovascular variables such as blood pressure and blood lipids. There are few studies that examined the effects of high intensity circuit training in young elderly overweight individuals. One recent paper by Romero-Arenas investigated the effects of an high-resistance circuit training in an elderly population, showed a decrease of fat mass and an improvement in cardiovascular-related performance index but the author does not report any data about blood parameters [4]. The high-intensity interval circuit training is a technique that mix the concept of high-intensity interval training [47] with an high intensity resistance training technique [8]. In our previous paper we have described the effects of this kind of training technique on some anthropometric and performance variables [19], the main point of strength of the HICT was the less time of effort and the greater effects on body composition and blood lipids whilst LICT seems to be more efficient in reducing DBP and ET in reducing total cholesterol. One caveat is that we have not measured the detraining effects to determine the duration the observed effects on blood lipids and blood pressure. It would therefore be appropriate to design also a detraining monitoring to assess the duration of the observed effects. Moreover it would be of interest to analyse blood lipids response to considered training methodologies in patients with severe hyperlipidaemia. Nevertheless our preliminary results are suggestive of a greater health effects promoted by an higher intensity circuit training.

## Competing interests

The authors declare that they have no competing interests.

## Authors’ contributions

AP and FP designed the study, performed the experiment, analysed the data and wrote the manuscript. TM evaluated the subjects and analysed the data. MN performed nutritional assessment and create the diet. GB participated in the design of the study and helped to draft the manuscript. GS and FB performed the body composition analysis and participated in the data analysis. AB made substantial contributions to the conception and design of the study. All authors read and approved the final manuscript.
